# Non-reciprocal Interspecies Hybridization Barriers in the Capsella Genus Are Established in the Endosperm

**DOI:** 10.1371/journal.pgen.1005295

**Published:** 2015-06-18

**Authors:** Carolin A. Rebernig, Clément Lafon-Placette, Marcelinus R. Hatorangan, Tanja Slotte, Claudia Köhler

**Affiliations:** 1 Department of Plant Biology, Uppsala BioCenter, Swedish University of Agricultural Sciences and Linnean Center of Plant Biology, Uppsala, Sweden; 2 Department of Ecology, Environment and Plant Sciences, Stockholm University, Stockholm, Sweden; Harvard University, UNITED STATES

## Abstract

The transition to selfing in *Capsella rubella* accompanies its recent divergence from the ancestral outcrossing *C*. *grandiflora* species about 100,000 years ago. Whether the change in mating system was accompanied by the evolution of additional reproductive barriers that enforced species divergence remained unknown. Here, we show that *C*. *rubella* and *C*. *grandiflora* are reproductively separated by an endosperm-based, non-reciprocal postzygotic hybridization barrier. While hybridizations of *C*. *rubella* maternal plants with *C*. *grandiflora* pollen donors resulted in complete seed abortion caused by endosperm cellularization failure, the reciprocal hybridization resulted in the formation of small seeds with precociously cellularized endosperm. Strikingly, the transcriptomic response of both hybridizations mimicked respectively the response of paternal and maternal excess hybridizations in *Arabidopsis thaliana*, suggesting unbalanced genome strength causes hybridization failure in both species. These results provide strong support for the theory that crosses between plants of different mating systems will be unbalanced, with the outcrosser behaving like a plant of increased ploidy, evoking a response that resembles an interploidy-type seed failure. Seed incompatilibity of *C*. *rubella* pollinated by *C*. *grandiflora* followed the Bateson-Dobzhansky-Muller model, involving negative genetic interaction of multiple paternal *C*. *grandiflora* loci with at least one maternal *C*. *rubella* locus. Given that both species only recently diverged, our data suggest that a fast evolving mechanism underlies the post-zygotic hybridization barrier(s) separating both species.

## Author Summary

Changes in mating system are supposed to change levels of sexual conflict, causing reduced conflict in inbreeding compared to outbreeding species. The recently diverged species pair of the inbreeding *Capsella rubella* and the outbreeding *C*. *grandiflora* provides the opportunity to test this hypothesis. While hybridizations of *C*. *rubella* maternal plants with *C*. *grandiflora* pollen donors gave rise to seeds with phenotypic similarities to paternal excess hybridizations in *Arabidopsis thaliana*, the reciprocal hybridization had similarities to maternal excess hybridizations. These results lend support to the hypothesis that selfing reduces sexual conflict, causing the outcrossing parent to have an increased effective ploidy compared to the selfing parent, resulting in unbalanced genome ratios after hybridization and seed failure. Seed failure correlates with either precocious or delayed endosperm cellularization, in agreement with the endosperm being the battleground for sexual conflict in flowering plants. *C*. *grandiflora* and *C*. *rubella* have only recently diverged, suggesting that the genes building the hybridization barrier are rapidly evolving as a consequence of sexual conflict.

## Introduction

The highly selfing species *Capsella rubella* separated about 100,000 years ago from the obligate outcrosser *C*. *grandiflora* [1,2]. While *C*. *rubella* is found throughout much of southern and western Europe, *C*. *grandiflora* is restricted primarily to the northwest of Greece [3,4]. The breakdown of self-incompatibility in Capsella was concurrent with species divergence [1,3,4], which was associated with a rapid loss of diversity in the newly founded *C*. *rubella* [5].

Changes in mating system have been proposed to change levels of sexual conflict, with parental conflict being less intense in self-pollinating plants than in outcrossers [6]. The sexual conflict theory implies that maternally and paternally inherited genes are not equal in relation to maternal investment in offspring, while paternally inherited genes promote maternal provisioning of the progeny, maternally inherited genes counteract this activity [7,8]. Based on this theory, if such genes evolved under different levels of selection pressure in two different species, hybridization should lead to unbalanced maternal investment to offspring, leading to the establishment of a postzygotic hybridization barrier.

Nonequivalence of maternal and paternal genomes can be explained by genomic imprinting, an epigenetic phenomenon causing differential expression of genes depending on their parent-of-origin [9]. In flowering plants, genomic imprinting plays a predominant role in the endosperm, a terminal nutritive tissue supporting embryo growth that is consumed by the embryo during seed development or after germination [10]. Most angiosperms follow a nuclear-type of endosperm development, where the endosperm initially develops as a syncytium and cellularization is triggered after a defined number of mitotic cycles [11,12]. Endosperm cellularization is a crucial developmental transition, which in case of failure triggers embryo arrest [13–15]. Studies on different plant species consistently reported interspecies hybrid seed lethality due to endosperm cellularization failure [7,16–21]. Similar endosperm defects have been observed in hybridizations of plants that differ in ploidy, suggesting a common mechanistic basis [13,21,22]. Specifically in Arabidopsis, hybridization between a maternal tetraploid plant and a paternal diploid plant results in precocious endosperm cellularization, while the reciprocal hybridization causes increased endosperm growth and delayed or failure of endosperm cellularization [13]. Interspecies postzygotic hybridization barriers that are established in the endosperm can often be bypassed by changing the ploidy of one parental species [23–25]. This suggests that these barriers have a quantitative basis and that endosperm-based hybridization barriers are a consequence of deregulated imprinted genes [26–28]. These theoretical considerations are supported by the imprinted gene *ADMETOS*, which is causally responsible for triploid seed abortion [29]. As parental conflict is predictably less intense in self-pollinating plants than in outcrossers [7], in hybridizations of plants with differing mating systems, outcrossing parents are expected to behave like plants of increased ploidy. Therefore, there should be symptoms of maternal excess when the outcrosser is the seed parent and symptoms of paternal excess in the reciprocal cross [6]. While endosperm phenotypes in response to interspecies crosses lent support for this hypothesis [6], molecular evidence has been lacking thus far. To close this knowledge gap, we have investigated the interspecies hybridization barrier between the closely related species pair *C*. *rubella* and *C*. *grandiflora* on a morphological, genetic, and molecular level. Our results provide strong support for the theory that crosses between plants of different mating systems will be unbalanced, with the outcrosser behaving like a plant of increased ploidy, evoking a response that resembles an interploidy-type seed failure.

## Results

### The postzygotic barrier between *C*. *rubella* and *C*. *grandiflora* non-reciprocally affects seed development

**Fig 1:**
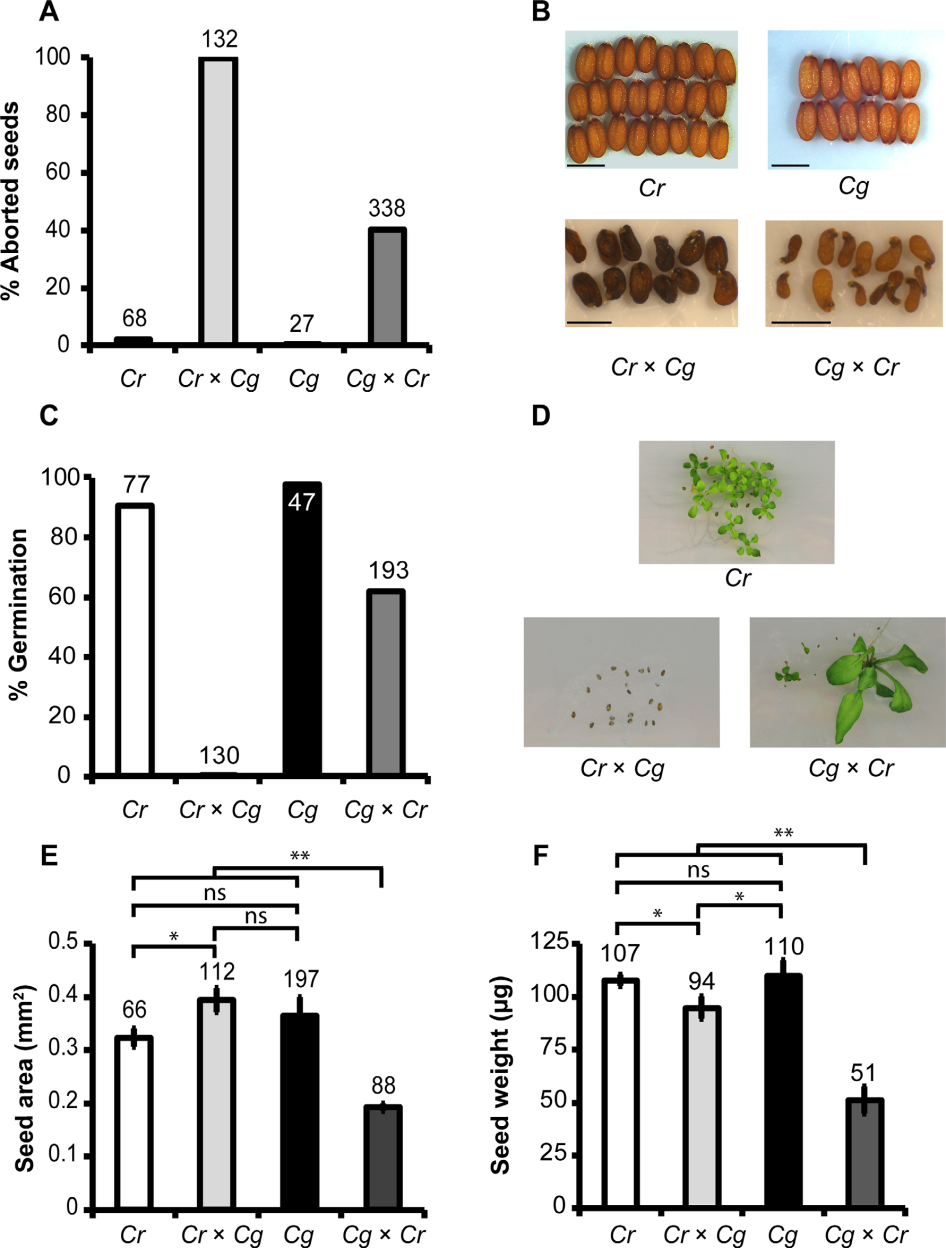
Cross direction-dependent incompatibility affects development of *Capsella rubella* and *C*. *grandiflora* hybrid seeds. Percentage (A) and phenotypes (B) of aborted and non-aborted seeds of *C*. *rubella*, *C*. *grandiflora* and reciprocal hybrids of both species. Scale bars reflect 1 mm. (C) Percentage of germinated seeds of indicated crosses. (D) Seedlings of indicated crosses 10 days after germination. Seed area (E) and seed weight (F) of indicated crosses. Error bars show standard deviation. Significance was determined by t test analysis. * P < 0.05, ** P < 0.01 ns, not significant. In all graphs numbers on top of the bars correspond to number of analyzed seeds.

The recently diverged *C*. *rubella* and *C*. *grandiflora* species are reproductively separated by different mating systems [1,3,4]. However, whether there are additional reproductive barriers between both species has not yet been investigated. We therefore performed reciprocal crosses between *C*. *grandiflora* and *C*. *rubella* and analyzed the resulting seed set. Pollinations in both directions were successful and resulted in similar numbers of seeds per silique between reciprocal crosses and intra-species crosses, revealing that there were no pollen incompatibilities between *C*. *rubella* and *C*. *grandiflora* (S1 Table). However, hybrid seeds developed abnormally and aborted at different frequencies; while about 40% of seeds after crosses of *C*. *grandiflora* × *C*. *rubella* (female × male) were abnormal (Fig 1A and 1B), corresponding to a germination rate of about 60% (Fig 1C), in the reciprocal cross all seeds aborted (Fig 1A, 1B and 1C). Hybrid seedlings of the cross *C*. *grandiflora* × *C*. *rubella* were variable in size and germination time point (Fig 1D) but developed into fertile adult plants. Intra-species crosses resulted in seed abortion rates below 5% and germination rates above 80% (Fig 1A and 1C). The seeds of the parental species were similar in size and weight (Fig 1B, 1E and 1F; *P* > 0.1, t-test). Seeds of the cross *C*. *rubella* × *C*. *grandiflora* were significantly larger than *C*. *rubella* seeds but lighter compared to both parental seeds (Fig 1E and 1F; *P* < 0.05, t-test), while seeds of the reciprocal cross were significantly smaller and lighter (Fig 1E and 1F; *P* < 0.01, t-test).

### Abortion of *C*. *rubella* × *C*. *grandiflora* hybrid seeds is a consequence of abnormal endosperm development

**Fig 2:**
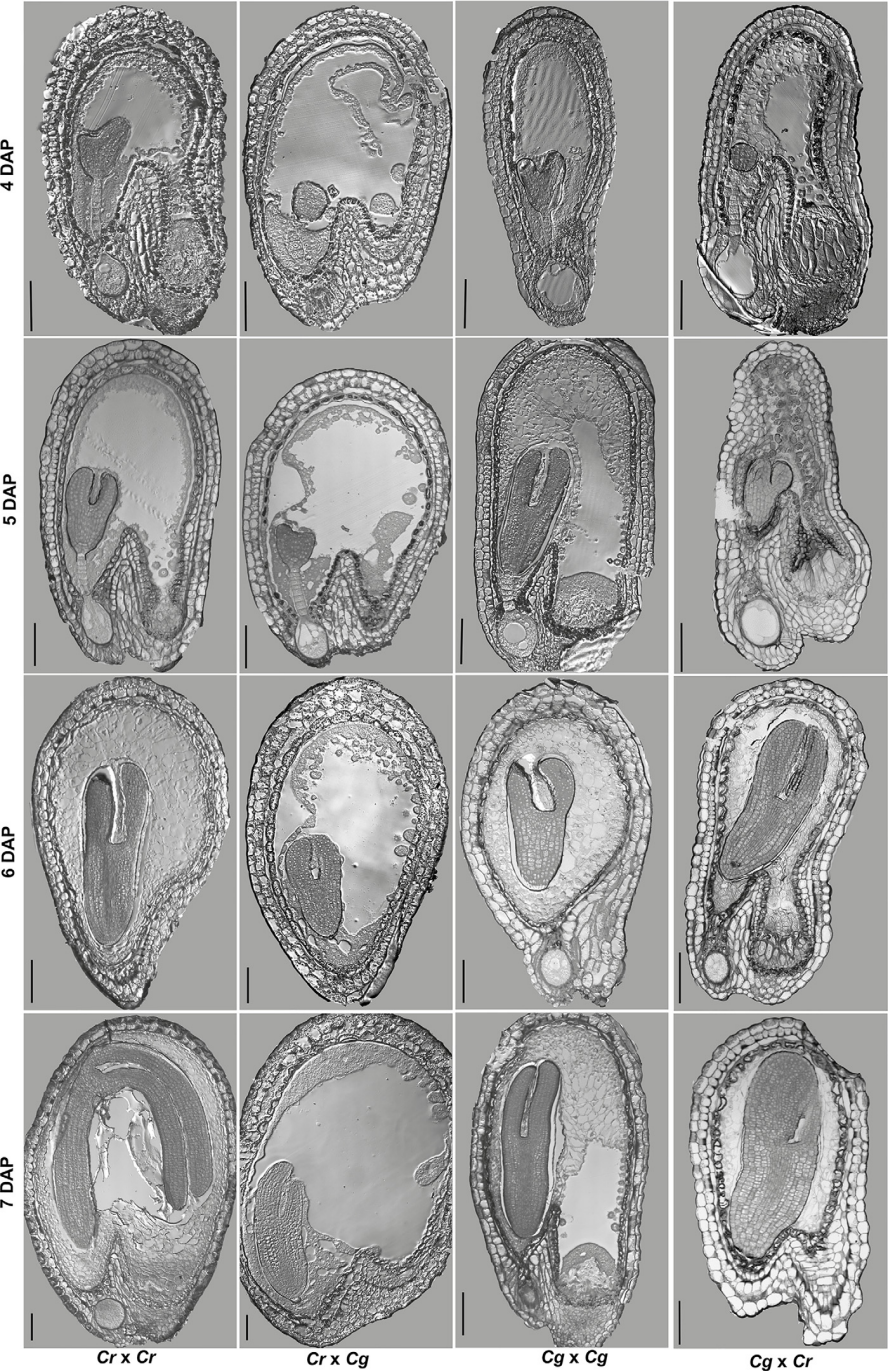
Hybrid seed incompatibility between *C*. *rubella* and *C*. *grandiflora* correlates with endosperm cellularization defects. Sections of *C*. *rubella*, *C*. *grandiflora* and reciprocal hybrid seeds at 4–7 days after pollination (DAP). Scale bar 100μm.

Detailed analysis of parental and hybrid seeds at defined time points (4–7 days after pollination, DAP) revealed differences in the timing of endosperm cellularization in hybrid seeds compared to parental seeds (Fig 2). In both parental species cellularization was completed at 6 DAP, with the embryo having reached the torpedo stage. In contrast, the endosperm of *C*. *rubella* × *C*. *grandiflora* hybrid seeds was not cellularized at 7 DAP and embryo development was substantially delayed (Fig 2). Conversely, in the reciprocal cross, cellularization happened precociously at 4 DAP and was completed at 5 DAP. The seed coat did not expand properly, leaving insufficient space for the embryo to bend.

**Fig 3:**
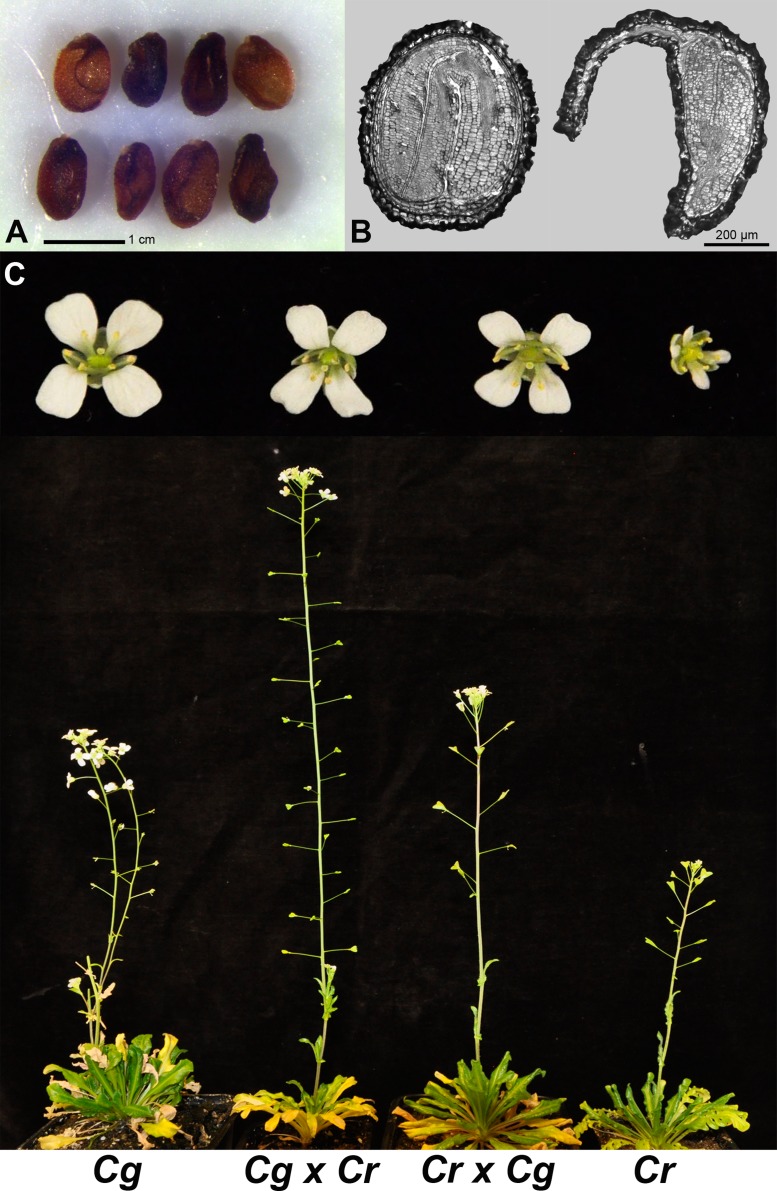
*C*. *rubella* × *C*. *grandiflora* hybrid embryos are viable, revealing a major role of endosperm defects in hybrid seed incompatibility. (A) *C*. *rubella* × *C*. *grandiflora* hybrid seeds at 13 days after pollination (DAP). (B) Section through seeds derived from crosses of *C*. *rubella* × *C*. *rubella* (left) and *C*. *rubella* × *C*. *grandiflora* (right) at 13 DAP. The sections reveal that hybrid embryos reach the torpedo stage at which development arrests. (C) Comparison of adult plants of all crosses (*C*. *grandiflora* × *C*. *grandiflora* (*Cg*), *C*. *grandiflora* × *C*. *rubella* (*Cg* × *Cr*), *C*. *rubella* × *C*. *grandiflora* (*Cr* × *Cg*), *C*. *rubella* × *C*. *rubella* (*Cr*)). Flower phenotypes of the respective genotypes are shown on top.

Delayed or complete block of endosperm cellularization has been correlated with embryo arrest [15]. We therefore tested whether abortion of hybrid *C*. *rubella* × *C*. *grandiflora* seeds is a consequence of abnormal endosperm development or rather caused by an embryo defect. To distinguish between both possibilities we isolated hybrid embryos at 13 DAP, when the endosperm was already completely collapsed, but the embryo was still viable (Fig 3A and 3B). Of 14 isolated embryos 9 developed normally and based on flower size the F1 plants were clearly recognized as hybrids (Fig 3C). *C*. *grandiflora* × *C*. *rubella* hybrids were easily obtained and resembled in flower size the reciprocal hybrid (Fig 3C). Together we conclude that abortion of *C*. *rubella* × *C*. *grandiflora* hybrid seeds is a consequence of abnormal endosperm development and most likely endosperm cellularization failure, which can be bypassed by embryo rescue.

### Endosperm cellularization failure is not coupled to proliferation abnormalities

**Fig 4:**
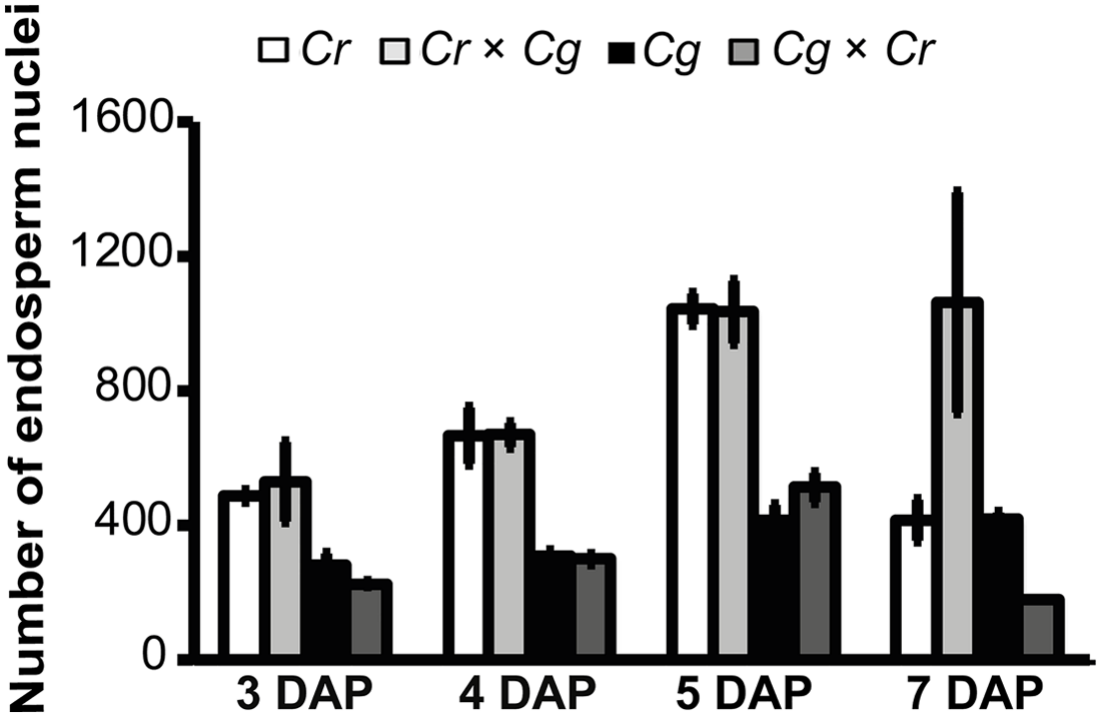
Hybrid seed incompatibility is not a consequence of endosperm proliferation defects. Endosperm nuclei numbers for each cross (*C*. *grandiflora* × *C*. *grandiflora* (*Cg*), *C*. *grandiflora* × *C*. *rubella* (*Cg* × *Cr*), *C*. *rubella* × *C*. *grandiflora* (*Cr* × *Cg*), *C*. *rubella* × *C*. *rubella* (*Cr*)) at indicated days after pollination (DAP). Three seeds per cross where counted. Error bars show standard error.

In interploidy crosses of *A*. *thaliana*, increased paternal ploidy causes increased endosperm proliferation and cellularization failure [13], mimicking crosses of *C*. *rubella* × *C*. *grandiflora*. Conversely, in seeds with increased maternal ploidy, endosperm proliferation is decreased and cellularization occurs precociously [13]. We therefore tested whether the endosperm cellularization defects in reciprocal Capsella crosses were reflected by changes in endosperm proliferation. Crosses of *C*. *rubella* × *C*. *grandiflora* resulted in similar proliferation rates compared to proliferation rates in the maternal *C*. *rubella* species, with nuclei numbers doubling between 3–5 DAP (Figs 4 and S1). Nuclei numbers decreased at 5–7 DAP in *C*. *rubella*, correlating with progression of embryo development. Failure of endosperm cellularization and arrest of embryo development correlated with unchanged nuclei numbers in *C*. *rubella* × *C*. *grandiflora* hybrid seeds between 5 and 7 DAP (Fig 4).

In the reciprocal cross, *C*. *grandiflora* × *C*. *rubella* nuclei proliferation followed that of the maternal *C*. *grandiflora* parent and nuclei numbers also doubled between 3–5 DAP. However, *C*. *grandiflora* as well as *C*. *grandiflora* x *C*. *rubella* hybrid seeds had only half the nuclei numbers compared to *C*. *rubella* and *C*. *rubella* x *C*. *grandiflora* seeds, revealing different endosperm proliferation rates between both parental species. At 7 DAP nuclei numbers in *C*. *grandiflora* x *C*. *rubella* seeds were only half that compared to *C*. *grandiflora* seeds, which is likely a consequence of increased endosperm consumption by the hybrid embryo. Together, we conclude that endosperm proliferation in the hybrids followed that of their maternal parental species, suggesting that the hybridization block caused by failure in endosperm cellularization is not a consequence of abnormal endosperm proliferation.

### Interspecies and interploidy hybridizations cause similar molecular defects

**Fig 5:**
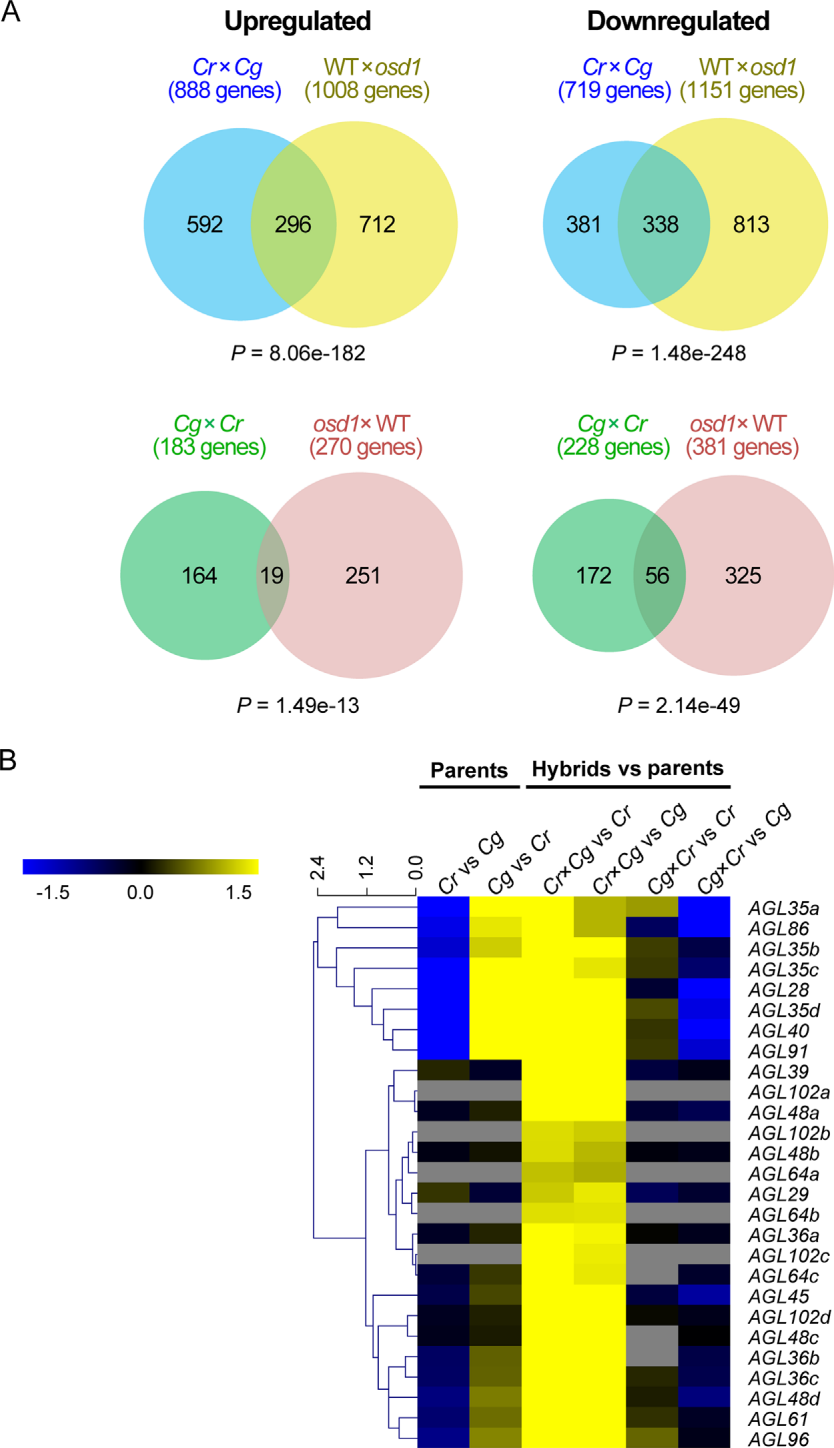
Molecular response to reciprocal hybridizations of *C*. *rubella* × *C*. *grandiflora* is similar to interploidy hybridizations in Arabidopsis. (A) Genes up- and downregulated in *C*. *rubella* × *C*. *grandiflora* reciprocal hybrid seeds compared to both parents overlap with genes deregulated in Arabidopsis interploidy seeds. The Arabidopsis *osd1* mutant produces unreduced gametes, mimicking an interploidy hybridization when crossed with wild-type (WT). P values reflecting significance of overlap were calculated using a hypergeometric test. (B) Heatmap of expression log2 fold changes of selected *AGL* genes between samples. Capsella *AGLs* with several homologs in Arabidopsis are marked by small letters.

The observed non-reciprocal defects in Capsella hybrid seeds resembled defects in response to interploidy hybridizations in Arabidopsis [13]. To test whether the similarity at the phenotypic level was also reflected at the molecular level, we generated genome-wide expression data of *C*. *rubella* and *C*. *grandiflora* parental seeds and reciprocal hybrid seeds. We filtered for genes exhibiting transgressive expression towards both parents, thus having either increased or decreased expression levels compared to both parents. For each gene we identified the closest homolog in Arabidopsis and analyzed the expression of those genes in interploidy hybrid seeds generated using the *omission of second division1* (*osd1*) mutant [30,31]. Loss of *OSD1* causes the formation of unreduced male and female gametes at high frequency [32], allowing to mimic interploidy hybridizations. A large proportion of genes were similarly deregulated in *C*. *rubella* × *C*. *grandiflora* hybrid seeds and seeds of paternal excess hybridizations in Arabidopsis (Fig 5A, S2 Table), while substantially fewer genes overlapped with deregulated genes in maternal excess seeds (S2 Fig). Conversely, in the reciprocal cross a substantial number of downregulated genes overlapped with downregulated genes in maternal excess hybridizations in Arabidopsis (Fig 5A, S2 Table), whereas the overlap with deregulated genes in paternal excess seeds was less pronounced (S2 Fig). These data reveal that interspecies and interploidy hybridizations cause a similar molecular response; while *C*. *rubella* × *C*. *grandiflora* hybrid seeds mimic a paternal excess phenotype, *C*. *grandiflora* × *C*. *rubella* hybrid seeds mimic a maternal excess phenotype. Notably, genes related to microtubular activity were enriched among downregulated genes in both *C*. *rubella* × *C*. *grandiflora* and Arabidopsis paternal excess hybrid seeds, while upregulated genes were enriched for genes involved in cell wall modification and specifically in glycosyl hydrolyzing activity (S3 Table). Microtubules are required in the phragmoplast for the transport of vesicles to construct a new cell wall [33], while glycosyl hydrolyzing enzymes can degrade pectin, which is assumed to be a key step in the deconstruction of plant cell walls [34]. Therefore, reduced expression of genes that are potentially required to build the phragmopast together with increased expression of genes that degrade cell walls correlates with the observed cellularization failure in interploidy and interspecies hybrid seeds (S3 Table). Conversely, in the reciprocal cross *C*. *grandiflora* × *C*. *rubella*, pectinesterase encoding genes were significantly downregulated, suggesting that inhibition of pectin degradation promotes cellularization.

Increased expression of type I MADS-box *AGAMOUS-LIKE* (*AGL*) gene*s* has been functionally connected to interploidy and interspecies seed arrest [15,25,35–37]. To test whether the same concept applies to Capsella interspecies hybrids, we isolated type I *AGL*s in the *C*. *rubella* genome according to the Phytozome annotation (S4 Table). Consistently, we identified a large number of *AGL*s being upregulated in *C*. *rubella* × *C*. *grandiflora* seeds compared to both parents (Fig 5B, S4 Table) and confirmed these results for selected *AGLs* (homologs of *AGL28*, *AGL36a*, *AGL61*) in independent experiments (S3 Fig). *AGL62* and *PHE1* have been functionally connected to interspecies seed abortion [25,35], however, read numbers were too low to detect significant expression changes for either gene. Nevertheless, by qPCR analysis we could detect strongly increased expression levels of both genes in *C*. *rubella* × *C*. *grandiflora* hybrid seeds (S3 Fig). We also tested expression of *ADM*, which is causally responsible for triploid seed abortion in Arabidopsis [29]. However, increased *ADM* expression in *C*. *rubella* × *C*. *grandiflora* hybrid seeds was only detected quite late during seed development at 7 DAP (S3 Fig), making it unlikely that increased *ADM* expression is causally responsible for interspecies seed arrest.

### Multiple genetic loci control incompatibility of *C*. *rubella* × *C*. *grandiflora* hybrid seeds

**Fig 6:**
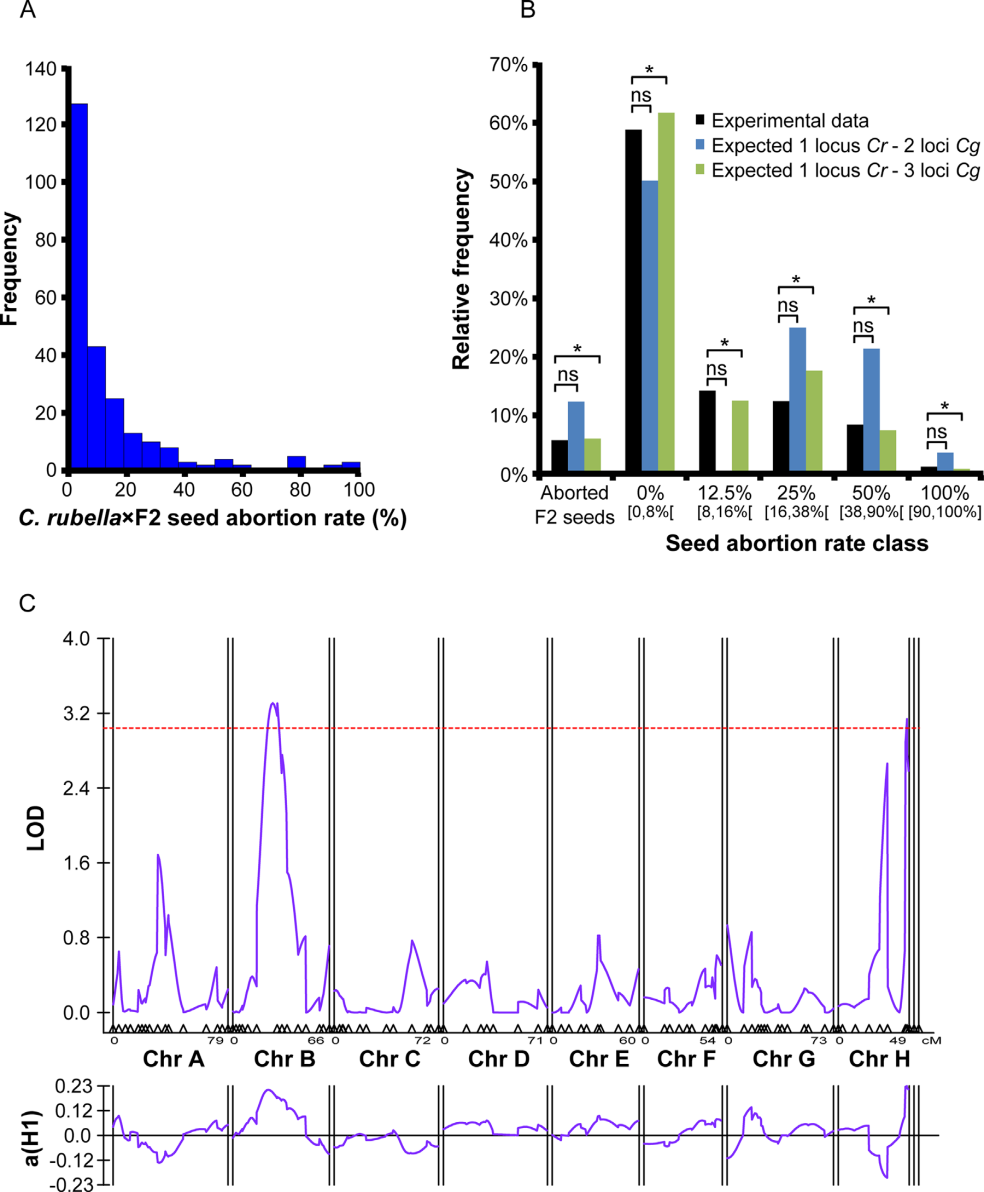
*C*. *rubella* × *C*. *grandiflora* hybrid seed incompatibility involves multiple genetic loci. (A) Frequency of F2 plants derived from crosses of *C*. *grandiflora* × *C*. *rubella* producing defined rates of seed abortion when backcrossed to *C*. *rubella* maternal plants. (B) Theoretical distribution of F2 plants producing defined rates of seed abortion when assuming that one maternal locus from *C*. *rubella* negatively interacts with two or three paternal loci from *C*. *grandiflora*. Experimental data correspond to data shown in (A). Significance of the observed distributions with the predictions of the models has been tested by Chi-square and P>0.05 is marked by an asterisk. Ns, non significant. (C) LOD scores for paternal *C*. *grandiflora* QTLs affecting abortion of *C*. *rubella* × *C*. *grandiflora* hybrid seeds. The purple line (top) represents the LOD score of each marker and the purple line (bottom) represents the effects. The red line represents the significance threshold as estimated by 1,000 permutations.

Seeds derived from selfed F1 plants aborted at a frequency of 5.7% (n = 7 plants, total number of seeds = 207), suggesting a multilocus genetic interaction. To estimate the number of loci involved in postzygotic hybrid incompatibility in the cross combination *C*. *rubella* × *C*. *grandiflora* we generated an F2 population of plants from selfed F1 individuals and backcrossed them as pollen donors to *C*. *rubella* plants (*C*. *rubella* × F2). We developed a genetic model assuming that one *C*. *rubella* maternal locus negatively interacts with two or three *C*. *grandiflora* paternally derived loci in a Bateson–Dobzhansky–Muller-type interaction, whereby divergent alleles at two or more loci negatively interact in hybrids to reduce fitness [38,39] (S4 Fig, S5 Table). Thus, based on this model hybrid seed abortion only occurs when two or three unlinked *C*. *grandiflora* paternal loci are inherited together. The frequency distribution of F2 plants (n = 250) triggering specific seed abortion rates in the backcrosses to *C*. *rubella* (Fig 6A) fitted the prediction of the model with three *C*. *grandiflora* paternal loci negatively interacting with one maternal *C*. *rubella* locus (Fig 6B), revealing a multilocus negative genetic interaction underlying hybrid incompatibility between *C*. *rubella* and *C*. *grandiflora*.

To identify the paternal *C*. *grandiflora* genetic loci contributing to postzygotic hybrid incompatibility with a *C*. *rubella* seed parent, we determined seed abortion rates after crossing a RIL population of *C*. *grandiflora* / *C*. *rubella* [40] to *C*. *rubella* maternal plants (S5A Fig). The parental accessions of the RIL population gave rise to 100% seed abortion in the *C*. *rubella* × *C*. *grandiflora* cross and 50% seed abortion in the reciprocal cross (S5B Fig).

The majority of RILs did not trigger seed abortion (S5A Fig), suggesting that hybrid incompatibility genes are purged from this population. Nevertheless, we could identify two significant QTLs located on chromosomes 2 and 8 (Fig 6C) that showed a positive correlation between *C*. *grandiflora* alleles and seed abortion. Both QTLs explain about 30% of the phenotypic variance, supporting the idea that there are more than two genes involved in conferring hybrid seed abortion and that incompatibility genes likely have been lost in the RIL population.

## Discussion

Recent divergence of *C*. *rubella* and *C*. *grandiflora* has been associated with the loss of self-incompatibility in *C*. *rubella* [1,2]. This drastic change in mating system most probably decreased gene flow between the two species, increasing genetic divergence and allowing additional reproductive barriers to be established. Our data lend support to this hypothesis by revealing a cross direction-dependent, non-reciprocal effect on hybrid seed development. Hybrid seeds resulting from *C*. *rubella* × *C*. *grandiflora* hybridizations were completely unviable; while the other cross direction resulted in smaller seeds that however were partially viable. Similar examples of unidirectional hybrid incompatibility were previously reported [6,41,42] and have been conceptionalized in the "weak inbreeder/strong outbreeder" (WISO) hypothesis [6]. Based on this hypothesis, in crosses between plants with differing mating systems outcrossing parents are expected to cause a stronger detrimental effect on seed development than selfing parents, as parental conflict is less intense in self-pollinating plants than in outcrossers [6]. Our data provide strong support for this hypothesis, suggesting that genes evolving under parental conflict may build the interspecies hybridization barrier between *C*. *rubella* and *C*. *grandiflora*. In angiosperms, the endosperm is the battleground for parental conflict [7], consistent with our data revealing that the endosperm is causally responsible for interspecies seed arrest. In *C*. *rubella* × *C*. *grandiflora* hybrid seeds, endosperm cellularization fails, which likely causes embryo arrest [15]. Supporting this idea, viable hybrids of *C*. *rubella* × *C*. *grandiflora* could easily be generated using *in vitro* embryo rescue. Conversely, arrest of *C*. *grandiflora × C*. *rubella* hybrid seeds is likely caused by precocious endosperm cellularization, in agreement with data showing that precocious endosperm cellularization causes decreased seed size and in extreme cases seed arrest [13,43]. Similar to data from rice interspecies hybrids revealing that endosperm cellularization and endosperm proliferation are uncoupled [21], we did not detect obvious endosperm proliferation defects that could explain differences in endosperm cellularization. Therefore, the observed correlation between precocious or delayed endosperm cellularization with respectively decreased or increased endosperm proliferation in response to interploidy hybridizations [13,44] seems unlikely to reflect a general mechanism coupling endosperm proliferation to cellularization. Our transcriptome data revealed that endosperm cellularization failure in response to interspecies and interploidy hybridizations correlated with increased expression of enzymes that hydrolyze glycosidic bonds between carbohydrates and could therefore degrade cell walls [45]. This suggests that the timing of endosperm cellularization is transcriptionally controlled and requires suppression of cell wall degrading enzymes.

Our data suggest that there are three or more *C*. *grandiflora* paternal loci that confer seed abortion in combination with a *C*. *rubella* seed parent. Similarly, the hybridization barrier between *A*. *thaliana* and *A*. *arenosa* is controlled by many genes participating in a complex genetic network [46], suggesting that postzygotic hybrid incompatibility that manifests in the endosperm is built by rather complex genetic interactions. Consistent with a scenario that there are many small effect genes contributing to hybrid seed abortion in Capsella, the identified QTLs explained only a low percentage of the phenotypic variation. Nevertheless, it is possible that major QTLs contributing to hybrid seed lethality have been purged from the RIL population and only small effect QTLs are maintained. The Bateson-Dobzhansky-Muller (BDM) model provides a theoretical framework explaining postzygotic reproductive isolation and substantial evidence in support of this model have accumulated over recent years [47–49]. This model stipulates that the interaction of independently evolving loci is innocuous in their native genomic context, however, the interaction between them is deleterious in hybrids [38,39]. Several studies in multiple plant species indicate that the genetic basis of reproductive isolation may involve relatively few loci [49–52]. This is contrasted by other studies supporting a scenario of multiple small incompatibilities accumulating over time [46,53–56]. Our study lends support to the latter, suggesting that multiple small incompatibilities can quickly evolve. A role of imprinted genes in building the interspecies hybridization barrier could provide an explanation for the fast evolution of hybridization barriers.

In conclusion, this study supports the “weak inbreeder/strong outcrosser” hypothesis [6] and suggests that a fast evolving, multi-locus mechanism underlies the post-zygotic barrier affecting *C*. *rubella* × *C*. *grandiflora* seed survival.

## Material and Methods

### Plant material and growth conditions

All seeds were surface sterilized using 5% Sodium Hypochlorite solution under the fume hood. After sterilization seeds were plated on MS media containing 1% sucrose. After stratification for two days in the dark at 4°C seedlings were grown in a growth room under long-day photoperiod (16 hrs light and 8 hrs darkness) at 22°C light and 20°C darkness temperature and a light intensity of 110 μE. All seedlings were transferred to pots and plants were grown in a growth chamber at 60% humidity and daily cycles of 16 hrs light at 21°C and 8 hrs darkness at 18°C.

For all crosses, designated female partners were emasculated, and the pistils were hand-pollinated 2 days after emasculation (same procedure for selfing and outcrossing plants).

Capsella accessions Cr48.21, Cg88.14 and Cg4a were used for the phenotypic analysis and Cr48.21 and Cg4a for the gene expression and QTL analyses. All accessions originated from wild collections. An F2 hybrid population of *C*. *grandiflora* × *C*. *rubella* individuals was generated based on an F1 interspecies cross (*C*. *grandiflora × C*. *rubella;* accessions: Cg4a × Cr48.21). F1 hybrid plants were selfed and the resulting F2 seeds were grown. Capsella QTL lines were described in [40]. Hybrid embryos derived from seeds of crosses of *C*. *rubella* (Cr48.21) × *C*. *grandiflora* (Cg4a) were isolated at 13 days after pollination (DAP). After a short incubation of siliques in 70% ethanol, the embryo was isolated using fine needles and placed on MS medium containing 2% sucrose. Plates were covered with a tissue and incubated in a growth room under conditions as indicated above. Surviving seedlings were transferred to soil.

### Seed size and seed weight analysis

Seeds were arranged on white plastic dishes and pictures were taken using a Leica Z16apoA microscope. Images were converted to black and white using the ‘‘threshold” function in ImageJ (http://rsbweb.nih.gov/ij/) and seed size was measured using the ‘‘Analyze Particles” function. For seed weight, 30–150 seeds per replicate (three replicates per cross) were weighed with an Ohaus GA110 balance and the total weight was divided by the number of seeds.

### Microscopy

Clearing analysis was performed as previously described [57].

Endosperm nuclei counts were conducted on cleared seeds at defined time points. After one day incubation in chloral hydrate solution (glycerol/chloral hydrate/water in a ratio of 1:8:3) at 4°C pictures in all possible planes of the seed were taken and endosperm nuclei were subsequently counted in all possible seed planes. Endosperm nuclei were counted using Adobe Photoshop CS5. For tissue sections, seeds were fixed and embedded with Technovit 7100 (Heraeus Kulzer, Hanau, Germany) following the procedure described in [15]. Treatment of the embedded seeds was conducted following [30]. Pictures of embedded and cleared samples were taken using a Leica DMI 4000B microscope and Leica DFC360 FX camera. Pictures of mature seeds were taken using a Leica Z16apoA microscope and a Leica DFC425C camera. All pictures were processed using Adobe Photoshop CS5.

### RNA sequencing and expression analysis

Two biological replicates with 300–500 seeds per replicate were generated. Seeds were harvested at 6 DAP and stored at -20°C in RNAlater (Sigma-Aldrich, St Louis, USA). RNA extraction was done using RNAqueous Kit with Plant RNA Isolation Aid (Life Technologies, Carlsbad, USA). The RNA-sequencing libraries were prepared using TruSeq RNA Sample Preparation Kit v2 (Illumina, San Diego, USA) according to the manufacturer's instructions.

For quantitative RT-PCR analysis five siliques at each time point were harvested and frozen in liquid nitrogen (accessions: Cr48.21, Cg4a). Glass beads (1.25–1.55 mm) were added, and the samples were ground in a Silamat S5 (IvoclarVivadent, Ellwangen, Germany). RNA was extracted using the RNeasy Plant Mini Kit (Qiagen, Hilden, Germany) according to the manufacturer’s instructions. Residual DNA was removed using the RNase-free DNase kit (Qiagen) and cDNA was synthesized using the Fermentas First strand cDNA synthesis kit (Fermentas, Burlington, Canada) according to the manufacturer’s instruction.

Quantitative RT-PCR was performed using a Bio-Rad MyiQ single colour Real-Time PCR Detection System (BioRad, Hercules, USA) and Maxima SYBR green qPCR master mix (Fermentas) according to the manufacturer’s instruction. Specificity of the PCR reactions was confirmed by melting curve analysis (55°-95°C). Results were analyzed as described by [58] using *PP2A* as a reference gene (S6 Table).

### High-throughput RNA sequence analysis

Sequencing reads were aligned to the *C*. *rubella* genome v1.0 (Phytozome) using Stampy v1.0.23 [59]. Reads mapping to multiple locations were discarded using Samtools [60], by invoking view–q 4 option. The number of reads for each annotated gene were counted using the default option in htseq-count command from the HTSeq Python package [61] (S7 Table). Paired logarithmic fold changes between each replicate were calculated using the R/Bioconductor package DESeq2 [62]. Differentially regulated genes across the two replicates were detected using the rank product method [63] as implemented in the Bioconductor RankProd Package [64]. The test was run with 100 permutations and gene selection was corrected for multiple comparison errors using a *pfp* (percentage of false prediction) <0.05. Arabidopsis closest homologues of *C*. *rubella* genes were determined according to the Phytozome v1.0 annotation of the *C*. *rubella* genome. For overlaps between misregulated genes in Arabidopsis and Capsella, only genes having a homologue in the other species were kept. The significance of the overlap between deregulated genes was estimated using a hypergeometric test. Enrichment of GO categories was determined using ATCOECIS [65]. Homologues of *A*. *thaliana* type I *AGL*s were identified in the *C*. *rubella* genome according to the Phytozome *C*. *rubella* v1.0 genome annotation. *A*. *thaliana* type I *AGL*s were determined as in [66]. Sequencing reads are deposited as fastq files in the Gene Expression Omnibus (GSE67359).

### QTL analysis

The QTL analysis was done using Windows QTL Cartographer v2.5 [67]. The crossing type was set to selfing RILs line (Ri1). Genotype data were coded as follows: two (2) for homozygous *C*. *grandiflora* alleles, one (1) for heterozygous alleles, and zero (0) for homozygous *C*. *rubella* alleles. Marker information and linkage map were obtained from [40]. The number of RILs used in the analysis was 101. Composite Interval Mapping method (CIM) was applied by setting walking speed to 1 cM to recover maximum resolution (the average distance between markers is 6 cM). The background control parameter was set to standard model with backward regression to select any available possible QTLs with standard 5 control markers. Trait values to be tested were entered as binary value (1 for plants with >2% seed abortion and 0 for plants with <2% seed abortion). The 2% threshold was defined based on the log transformed distribution of seed abortion frequencies that showed two distinct distributions bordering at 2%. The LR significant threshold was determined by running 1000 permutation tests with p = 0.05.

### Pollen viability assay

Pollen viability among 20 F2 individuals was assessed using Alexander stain [68]. Collected inflorescences were incubated in 10% ethanol. The anthers were dissected on a slide prior to adding a few drops of Alexander stain. The samples were incubated at room temperature for 15 min and then examined under a Zeiss Axioplan microscope. Viable (pink) and aborted (green) pollen grains were counted for each sample (190–320 pollen grains per individual).

## Supporting Information

S1 Fig Seed clearings of hybrid and non-hybrid seeds that were used to determine endosperm nuclei numbers. Cleared seeds of the indicated crosses (*C*. *grandiflora* × *C*. *grandiflora* (*Cg*), *C*. *grandiflora* × *C*. *rubella* (*Cg* × *Cr*), *C*. *rubella* × *C*. *grandiflora* (*Cr* × *Cg*), *C*. *rubella* × *C*. *rubella* (*Cr*)) between 3 to 7 days after pollination (DAP). Scale bars correspond to 100 μM.
(TIF)

S2 Fig Molecular response to reciprocal hybridizations of *C*. *rubella* × *C*. *grandiflora*. Only few genes deregulated in *C*. *rubella* × *C*. *grandiflora* hybrid seeds compared to both parents overlap with deregulated genes in *osd1* × wild type (WT). Likewise, only few genes deregulated in *C*. *grandiflora* × *C*. *rubella* hybrid seeds compared to both parents overlap with deregulated genes in WT × *osd1*. The Arabidopsis *osd1* mutant produces unreduced gametes, mimicking an interploidy hybridization when crossed with WT. P values reflecting significance of overlap were calculated using a hypergeometric test.
(TIF)

S3 Fig Quantitative RT-PCR analysis of selected genes. Expression of indicated genes was tested at 4–7 days after pollination (DAP) in whole siliques derived from crosses of *Capsella rubella* × *C*. *rubella* (white bars), *C*. *rubella* × *C*. *grandiflora* (light grey bars), *C*. *grandiflora* × *C*. *grandiflora* (black bars), *C*. *grandiflora* × *C*. *rubella* (dark grey bars). In the cross *C*. *grandiflora* × *C*. *rubella* expression was undetectable at 6 and 7 DAP. Error bars represent standard deviation.
(TIF)

S4 Fig Genetic model predicting seed abortion rates in crosses of *C*. *rubella* × F2 plants (derived after selfing of an F1 plant *C*. *rubella* ×*C*. *grandiflora*). (A) Genetic model predicting a negative interaction between one maternal *C*. *rubella* locus (allele noted R) and three paternal *C*. *grandiflora* loci (alleles noted A, B and C) giving rise to seed lethality. The red color symbolizes the negative lethal interaction. Paternal *C*. *rubella* a, b, c alleles and maternal *C*. *grandiflora* r allele are compatible and result in viable seeds. (B) Viable F1 plants are generated by the cross *C*. *grandiflora* ♀ × ♂ *C*. *rubella*. Among F2 progeny produced from selfed F1s, those inheriting R (maternal) and ABC (paternal) will not survive. The surviving F2s are predicted to cause seed abortion when backcrossed to *C*. *rubella* (*C*. *rubella* ♀ × F2 ♂) according to the number of A, B, C alleles they inherited from *C*. *grandiflora*. Only the ABC loci combination in a paternal gamete will cause seed abortion by interacting with the R locus in *C*. *rubella* maternal plants.
(TIF)

S5 Fig Frequency of seed abortion in crosses of *C*. *rubella* × RILs and parental species of RILs. (A) Log2 transformed seed abortion distribution of *C*. *rubella* × RILs. (B) Seed abortion of crosses *C*. *grandiflora* × *C*. *rubella* (*Cg* ×*Cr*), *C*. *rubella* × *C*. *grandiflora* (*Cr* × *Cg*) using parental accessions of the RIL population.
(TIF)

S1 Table Seed number in inter- and intra-species crosses. (PDF)

S2 Table Genes up- and downregulated in *C*. *rubella* × *C*. *grandiflora* reciprocal hybrid seeds compared to both parents and genes deregulated in Arabidopsis interploidy seeds. (XLSX)

S3 Table Enriched GO terms of genes commonly deregulated in seeds of reciprocal *C*. *rubella* × *C*. *grandiflora* (*Cr* × *Cg*) hybridizations and Arabidopsis WT × *osd1* reciprocal interploidy hybridizations. (PDF)

S4 Table *Capsella rubella* homologues of Type I AGLs in *Arabidopsis thaliana*. (PDF)

S5 Table Genetic models predicting segregation of hybrid incompatibility in crosses of *C*. *rubella* × F2 plants (derived after selfing of an F1 plant *C*. *grandiflora* × *C*. *rubella*). (PDF)

S6 Table Primer sequences used for qPCR. (PDF)

S7 Table Quality of sequencing samples. (PDF)
