# Supplementary figures and images for: Non-reciprocal Interspecies Hybridization Barriers in the Capsella Genus Are Established in the Endosperm
Source: PLoS Genet. 2015 Jun 18;11(6):e1005295. doi: 10.1371/journal.pgen.1005295 (PMC4472357; doi:10.1371/journal.pgen.1005295)

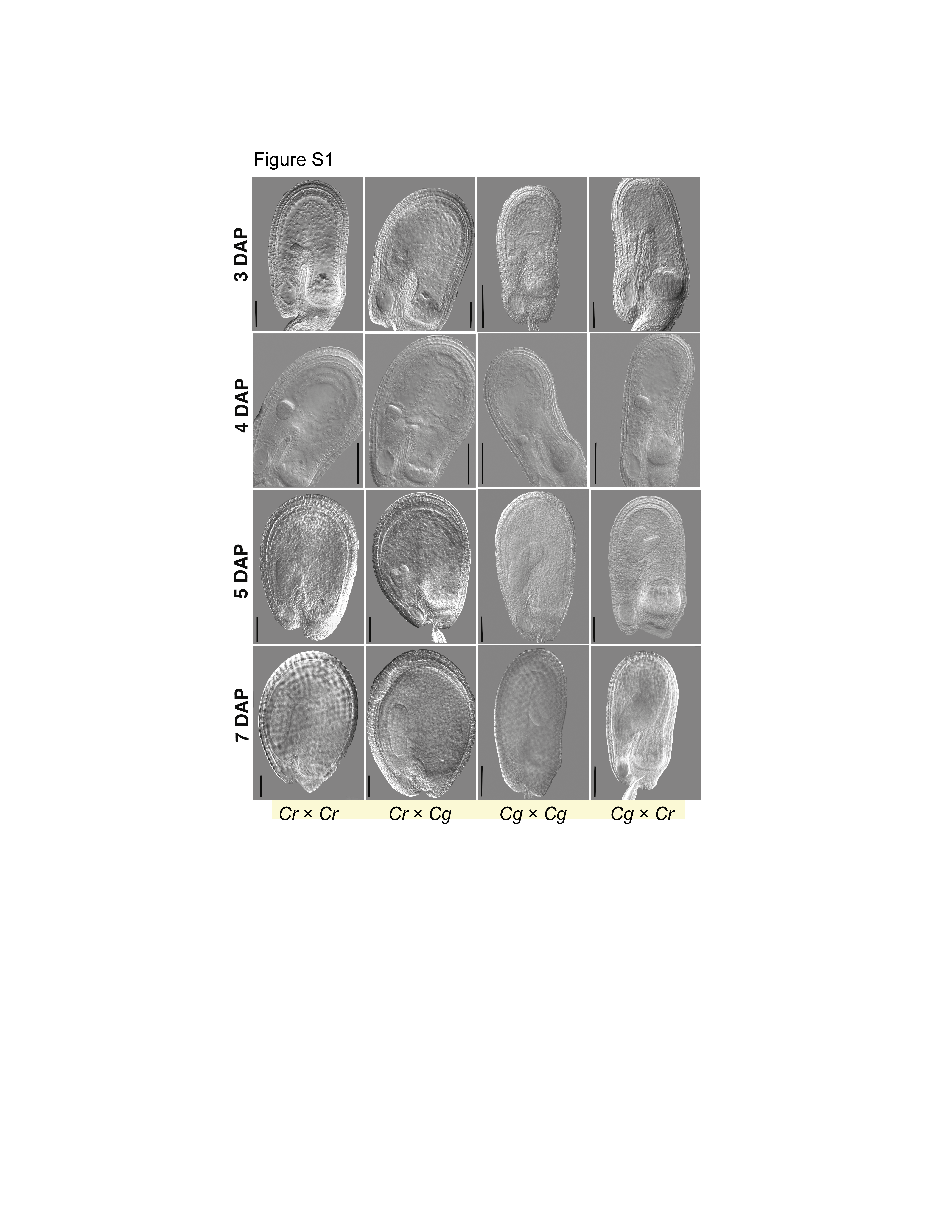

Supplement: S1 Fig — Cleared seeds of the indicated crosses (C. grandiflora × C. grandiflora (Cg), C. grandiflora × C. rubella (Cg × Cr), C. rubella × C. grandiflora (Cr × Cg), C. rubella × C. rubella (Cr)) between 3 to 7 days after pollination (DAP). Scale bars correspond to 100 μM. (TIF) [file pgen.1005295.s001.tif]

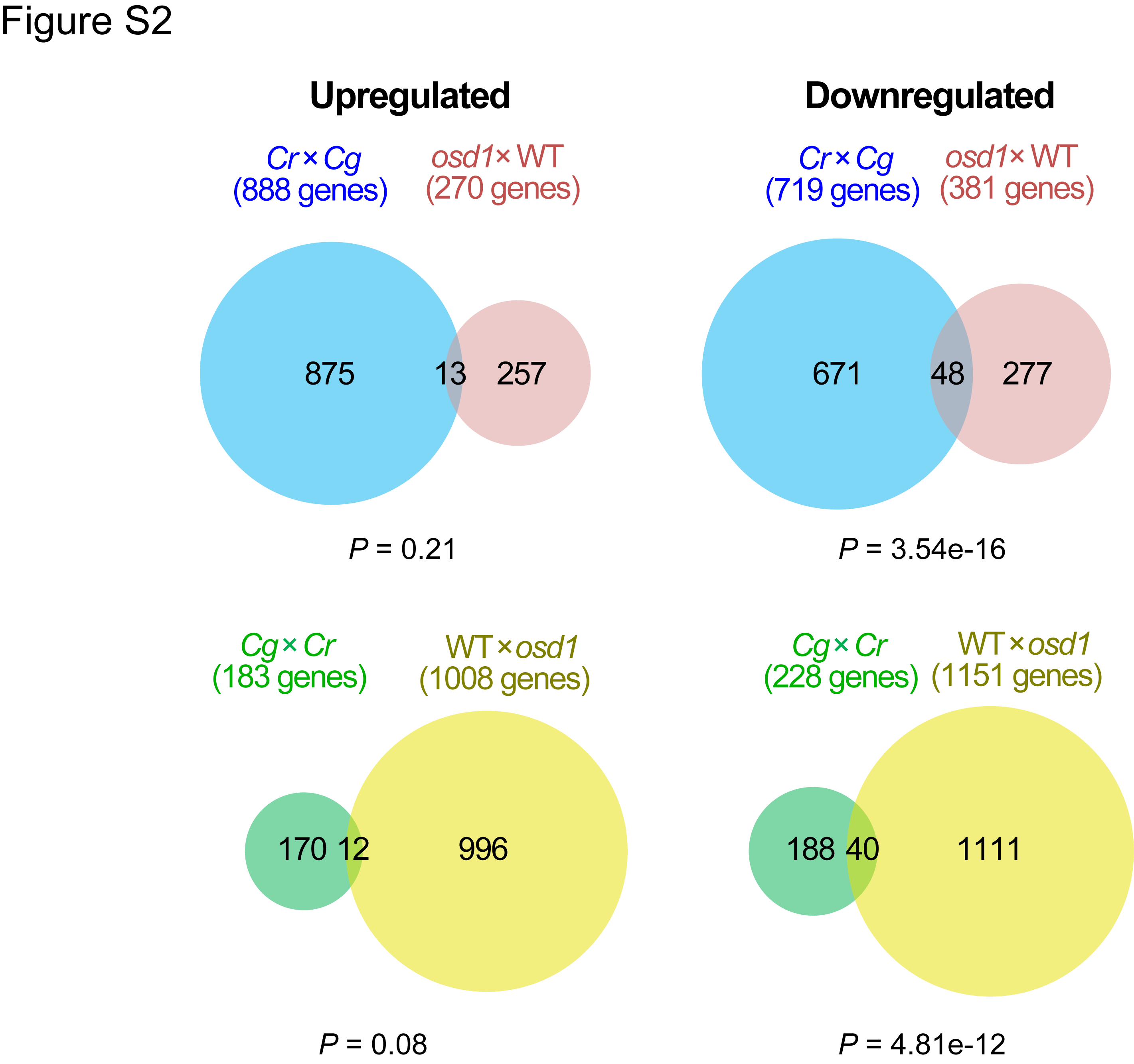

Supplement: S2 Fig — Only few genes deregulated in C. rubella × C. grandiflora hybrid seeds compared to both parents overlap with deregulated genes in osd1 × wild type (WT). Likewise, only few genes deregulated in C. grandiflora × C. rubella hybrid seeds compared to both parents overlap with deregulated genes in WT × osd1. The Arabidopsis osd1 mutant produces unreduced gametes, mimicking an interploidy hybridization when crossed with WT. P values reflecting significance of overlap were calculated using a hypergeometric test. (TIF) [file pgen.1005295.s002.tif]

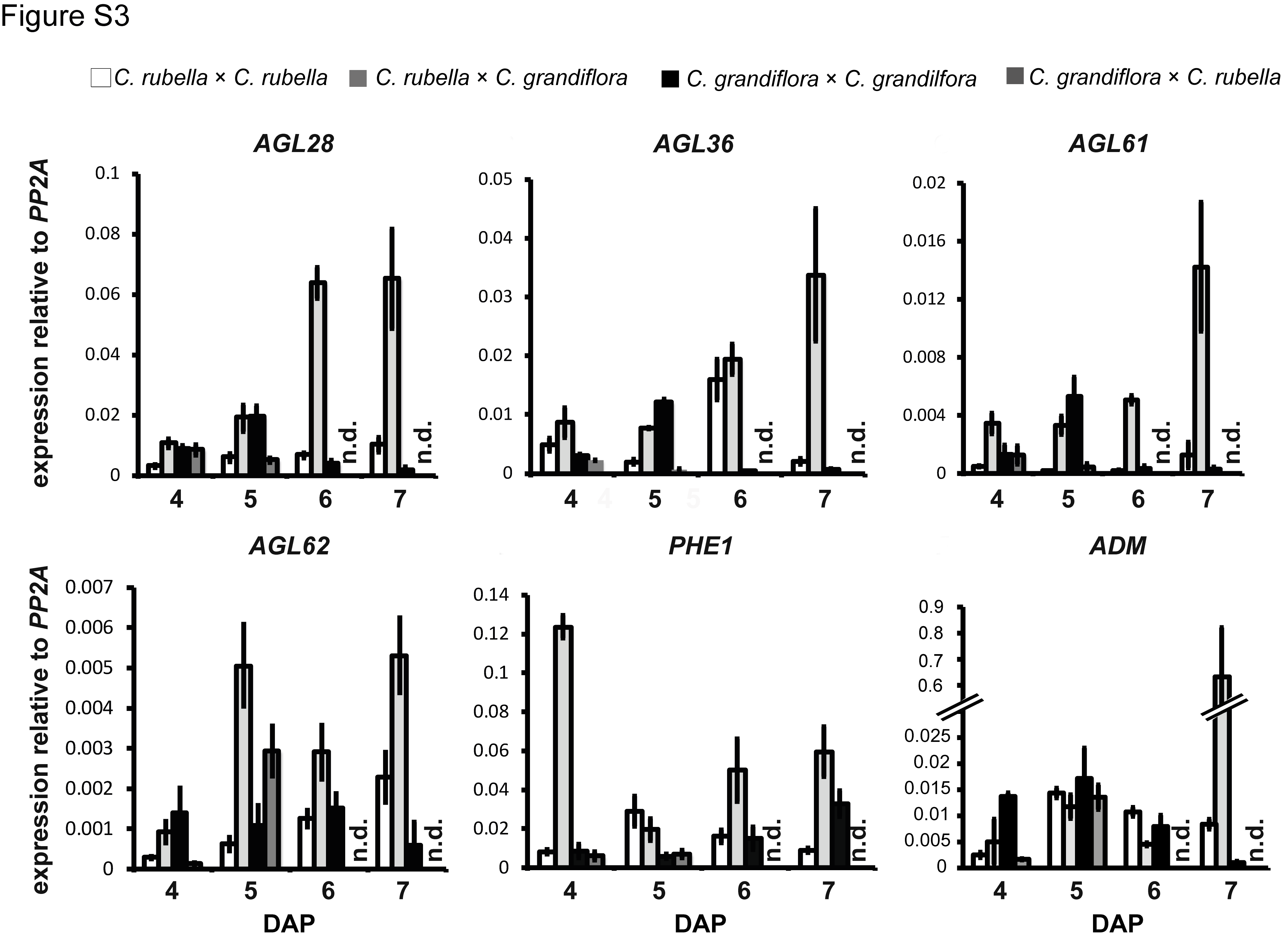

Supplement: S3 Fig — Expression of indicated genes was tested at 4–7 days after pollination (DAP) in whole siliques derived from crosses of Capsella rubella × C. rubella (white bars), C. rubella × C. grandiflora (light grey bars), C. grandiflora × C. grandiflora (black bars), C. grandiflora × C. rubella (dark grey bars). In the cross C. grandiflora × C. rubella expression was undetectable at 6 and 7 DAP. Error bars represent standard deviation. (TIF) [file pgen.1005295.s003.tif]

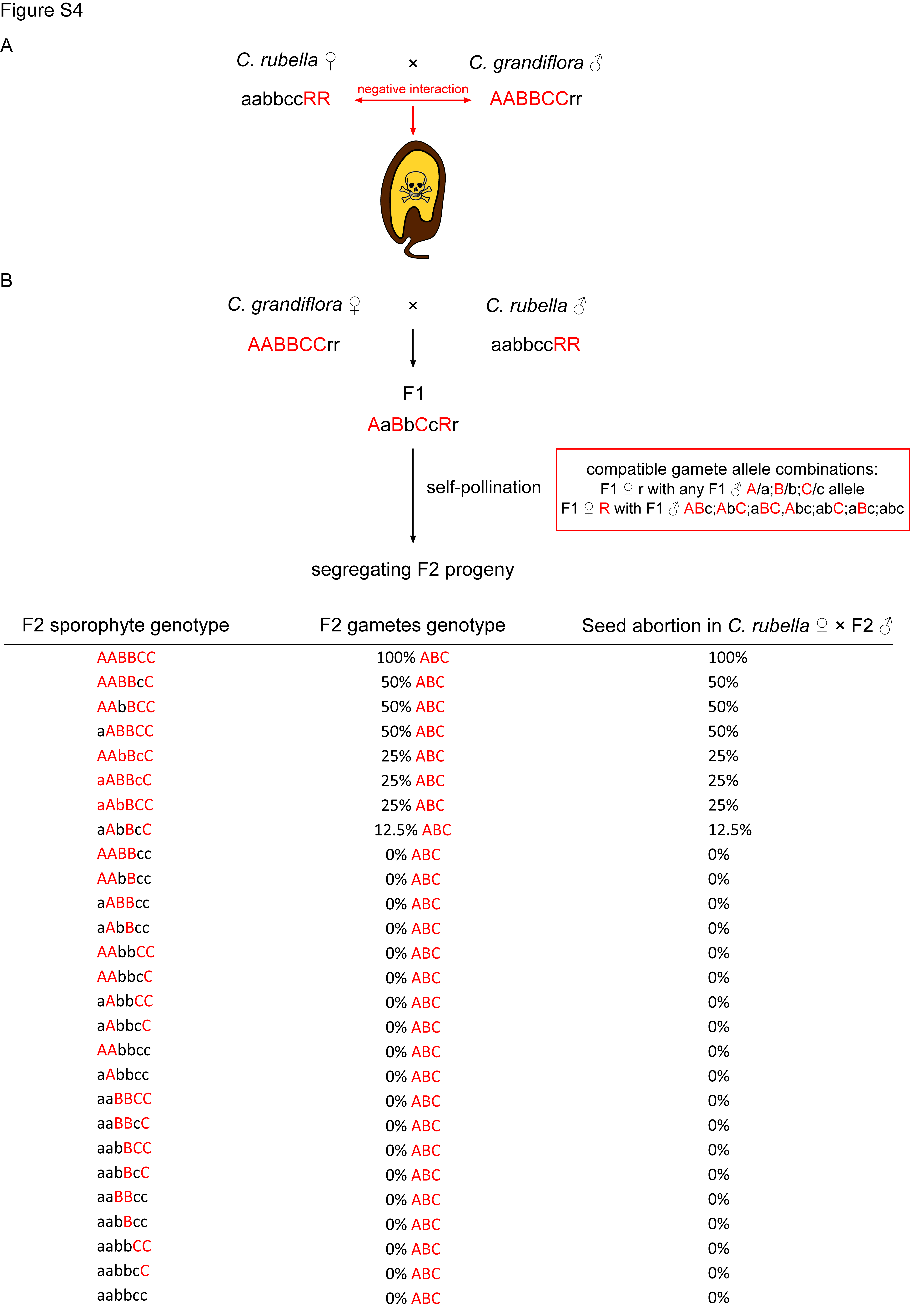

Supplement: S4 Fig — (A) Genetic model predicting a negative interaction between one maternal C. rubella locus (allele noted R) and three paternal C. grandiflora loci (alleles noted A, B and C) giving rise to seed lethality. The red color symbolizes the negative lethal interaction. Paternal C. rubella a, b, c alleles and maternal C. grandiflora r allele are compatible and result in viable seeds. (B) Viable F1 plants are generated by the cross C. grandiflora ♀ × ♂ C. rubella. Among F2 progeny produced from selfed F1s, those inheriting R (maternal) and ABC (paternal) will not survive. The surviving F2s are predicted to cause seed abortion when backcrossed to C. rubella (C. rubella ♀ × F2 ♂) according to the number of A, B, C alleles they inherited from C. grandiflora. Only the ABC loci combination in a paternal gamete will cause seed abortion by interacting with the R locus in C. rubella maternal plants. (TIF) [file pgen.1005295.s004.tif]

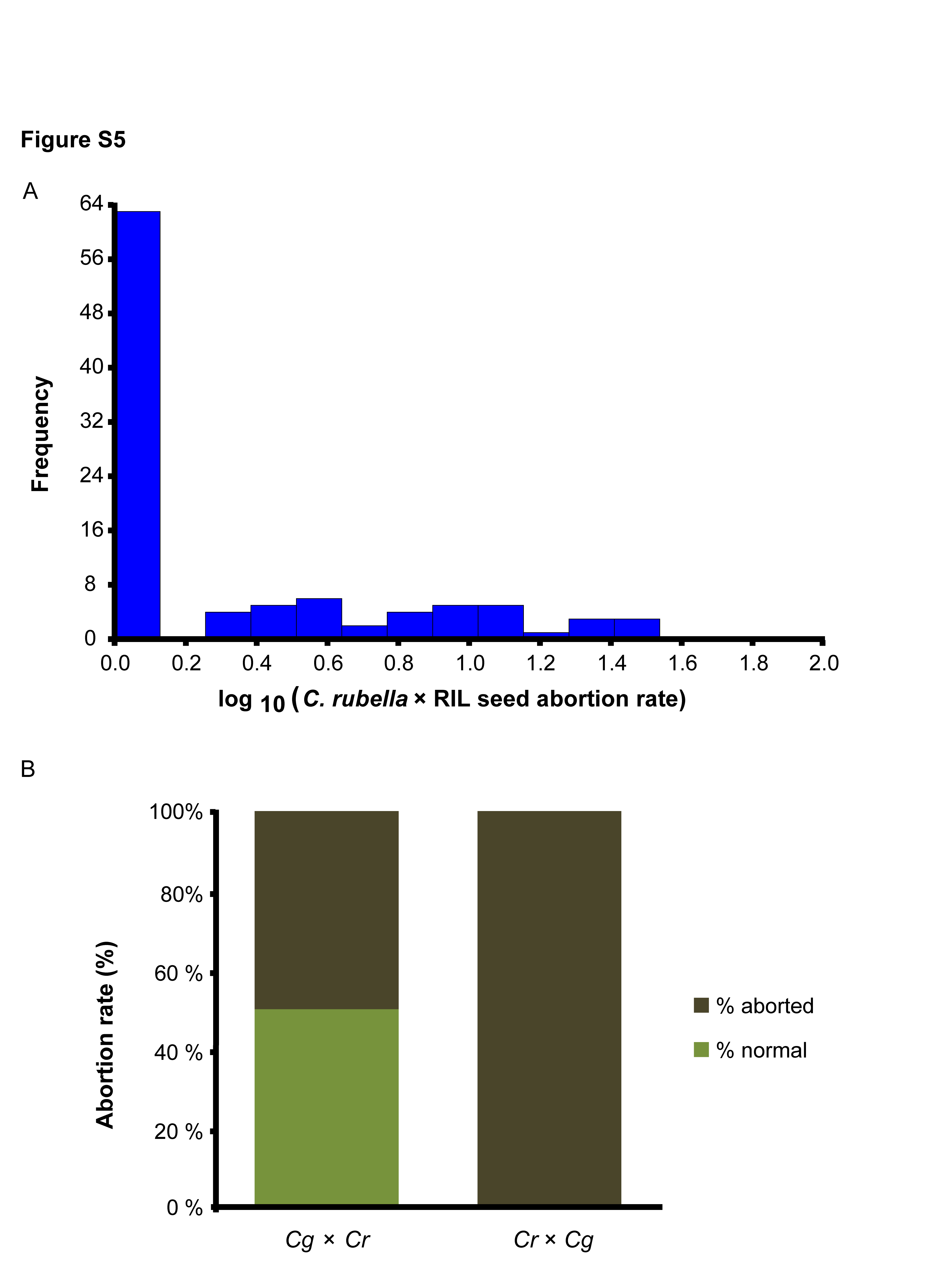

Supplement: S5 Fig — (A) Log2 transformed seed abortion distribution of C. rubella × RILs. (B) Seed abortion of crosses C. grandiflora × C. rubella (Cg ×Cr), C. rubella × C. grandiflora (Cr × Cg) using parental accessions of the RIL population. (TIF) [file pgen.1005295.s005.tif]
