# Supplementary material for: Non-reciprocal Interspecies Hybridization Barriers in the Capsella Genus Are Established in the Endosperm
Source: PLoS Genet. 2015 Jun 18;11(6):e1005295. doi: 10.1371/journal.pgen.1005295 (PMC4472357; doi:10.1371/journal.pgen.1005295)
Supplement: S1 Table — (PDF) [file pgen.1005295.s006.pdf]

S1 Table. Seed number in inter- and intra-species crosses.

**Genotypes**	**Siliques**	**Seeds**	**Seeds/Silique**
C. rubella Cr48.21 x *C. rubella* Cr48.21	40	341	8.525
C. rubella Cr48.21 x *C. grandiflora* Cg4a	44	370	8.409091
C. grandiflora Cg88.14 x *C. grandiflora* Cg4a	36	345	9.583333
C. grandiflora Cg4a x *C. rubella* Cr48.21	65	585	9
