# Supplementary material for: Non-reciprocal Interspecies Hybridization Barriers in the Capsella Genus Are Established in the Endosperm
Source: PLoS Genet. 2015 Jun 18;11(6):e1005295. doi: 10.1371/journal.pgen.1005295 (PMC4472357; doi:10.1371/journal.pgen.1005295)
Supplement: S3 Table — (PDF) [file pgen.1005295.s008.pdf]

S3 Table. Enriched GO terms of genes commonly deregulated in seeds of reciprocal *C. rubella* × *C. grandiflora* (*Cr* × *Cg*) hybridizations and Arabidopsis WT × *osd1* reciprocal interploidy hybridizations. GO terms are sorted by their *P*-value (lower to higher). Only GO categories with a *P*-value <0.001 are shown.

Commonly down-regulated genes in *Cr* × *Cg* and Arabidopsis WT × *osd1*

Molecular function

GO term	*P*-value	Number of genes	Description
GO:0003777	7.72E-14	14	F microtubule motor activity
GO:0003774	4.72E-12	14	F motor activity
GO:0019887	7.01E-10	9	F protein kinase regulator activity
GO:0019207	9.01E-10	9	F kinase regulator activity
GO:0030234	1.25E-07	16	F enzyme regulator activity
GO:0030528	1.21E-05	45	F transcription regulator activity
GO:0016563	2.29E-04	7	F transcriptional activator activity
GO:0003700	4.99E-04	37	F transcription factor activity

Biological process

GO term	*P*-value	Number of genes	Description
GO:0007018	1.41E-13	13	P microtubule-based movement
GO:0007017	1.04E-12	15	P microtubule-based process
GO:0030705	5.32E-12	13	P cytoskeleton-dependent intracellular transport
GO:0007010	1.11E-08	15	P cytoskeleton organization and biogenesis
GO:0051726	1.48E-08	11	P regulation of cell cycle
GO:0007049	4.83E-08	13	P cell cycle
GO:0046907	7.50E-07	19	P intracellular transport
GO:0051649	8.70E-07	19	P establishment of cellular localization
GO:0051641	9.70E-07	19	P cellular localization
GO:0051244	4.40E-06	46	P regulation of cellular physiological process
GO:0050794	5.00E-06	46	P regulation of cellular process
GO:0050791	8.78E-06	46	P regulation of physiological process
GO:0050789	1.02E-04	46	P regulation of biological process
GO:0006810	6.54E-04	46	P transport
GO:0006996	6.88E-04	17	P organelle organization and biogenesis
GO:0016043	7.31E-04	28	P cell organization and biogenesis
GO:0051234	7.64E-04	46	P establishment of localization
GO:0051179	8.29E-04	46	P localization

Cellular localization

GO term	*P*-value	Number of genes	Description
GO:0005875	5.79E-13	13	C microtubule associated complex
GO:0015630	3.67E-12	16	C microtubule cytoskeleton

GO:0044430	1.11E-10	16	C cytoskeletal part
GO:0005856	9.34E-10	16	C cytoskeleton
GO:0031225	1.65E-06	14	C anchored to membrane
GO:0009524	7.84E-04	4	C phragmoplast
GO:0005634	8.91E-04	46	C nucleus

Commonly up-regulated genes in *Cr* × *Cg* and Arabidopsis WT × *osd1*

**Molecular function**			
**GO term**	***P*-value**	**Number of genes**	**Description**
GO:0016798	1.96E-04	14	F hydrolase activity, acting on glycosyl bonds
GO:0004553	1.72E-04	13	F hydrolase activity, hydrolyzing O-glycosyl compounds

**Biological process**			
**GO term**	***P*-value**	**Number of genes**	**Description**
GO:0005975	8.00E-04	18	P carbohydrate metabolism
GO:0006355	5.36E-04	22	P regulation of transcription, DNA-dependent
GO:0006351	8.24E-04	22	P transcription, DNA-dependent

**Cellular localization**			
**GO term**	***P*-value**	**Number of genes**	**Description**
GO:0012505	1.20E-10	89	C endomembrane system

Commonly down-regulated genes in *Cg* × *Cr* and Arabidopsis *osd1* × WT

**Cellular localization**			
**GO term**	***P*-value**	**Number of genes**	**Description**
GO:0012505	1.52E-04	20	C endomembrane system

Commonly up-regulated genes in *Cg* × *Cr* and Arabidopsis *osd1* × WT

**Biological process**			
**GO term**	***P*-value**	**Number of genes**	**Description**
GO:0009415	8.48E-05	3	P response to water
GO:0009790	9.57E-05	4	P embryonic development
