# Supplementary material for: Non-reciprocal Interspecies Hybridization Barriers in the Capsella Genus Are Established in the Endosperm
Source: PLoS Genet. 2015 Jun 18;11(6):e1005295. doi: 10.1371/journal.pgen.1005295 (PMC4472357; doi:10.1371/journal.pgen.1005295)
Supplement: S4 Table — (PDF) [file pgen.1005295.s009.pdf]

S4 Table. *Capsella rubella* homologues of Type I *AGL*s in *Arabidopsis thaliana*. Type I *AGL*s in Arabidopsis were identified according to Bemer et al., 2010.

***C. rubella* gene identifier**	***A. thaliana* gene identifier**	***A. thaliana* gene name**
Carubv10017771m.g	*AT5G26880*	*AGL26*
Carubv10010156m.g	*AT1G01530*	*AGL28*
Carubv10025656m.g	*AT2G34440*	*AGL29*
Carubv10001920m.g	*AT5G26630*	*AGL35*
Carubv10002555m.g	*AT5G26630*	*AGL35*
Carubv10003757m.g	*AT5G26630*	*AGL35*
Carubv10008056m.g	*AT5G26630*	*AGL35*
Carubv10001166m.g	*AT5G26650*	*AGL36*
Carubv10003161m.g	*AT5G26650*	*AGL37*
Carubv10001714m.g	*AT1G65300*	*AGL38, PHE2*
Carubv10020903m.g	*AT1G65300*	*AGL38, PHE2*
Carubv10022335m.g	*AT1G65300*	*AGL38, PHE2*
Carubv10001421m.g	*AT5G27130*	*AGL39*
Carubv10007644m.g	*AT4G36590*	*AGL40*
Carubv10015167m.g	*AT3G05860*	*AGL45*
Carubv10024482m.g	*AT2G28700*	*AGL46*
Carubv10024493m.g	*AT2G28700*	*AGL46*
Carubv10024608m.g	*AT2G28700*	*AGL46*
Carubv10025317m.g	*AT2G28700*	*AGL46*
Carubv10027564m.g	*AT5G55690*	*AGL47*
Carubv10006793m.g	*AT2G40210*	*AGL48*
Carubv10022233m.g	*AT1G60040*	*AGL49*
Carubv10015981m.g	*AT2G40210*	*AGL49*
Carubv10016121m.g	*AT2G40210*	*AGL50*
Carubv10024813m.g	*AT2G40210*	*AGL51*
Carubv10025411m.g	*AT2G40210*	*AGL52*
Carubv10003642m.g	*AT5G27070*	*AGL53*
Carubv10021715m.g	*AT1G60920*	*AGL55*
Carubv10021738m.g	*AT1G60920*	*AGL56*
Carubv10016398m.g	*AT3G04100*	*AGL57*
Carubv10021825m.g	*AT1G72350*	*AGL60*
Carubv10024914m.g	*AT2G24840*	*AGL61, DIA*
Carubv10028580m.g	*AT5G60440*	*AGL62*
Carubv10002683m.g	*AT1G29962*	*AGL64*
Carubv10002712m.g	*AT1G29962*	*AGL64*
Carubv10002787m.g	*AT1G29962*	*AGL64*
Carubv10010430m.g	*AT1G29962*	*AGL64*
Carubv10010438m.g	*AT1G29962*	*AGL64*

Carubv10010833m.g	*AT1G29962*	*AGL64*
Carubv10011216m.g	*AT1G29962*	*AGL64*
Carubv10012214m.g	*AT1G29962*	*AGL64*
Carubv10012479m.g	*AT1G29962*	*AGL64*
Carubv10021362m.g	*AT1G29962*	*AGL64*
Carubv10021425m.g	*AT1G29962*	*AGL64*
Carubv10021446m.g	*AT1G29962*	*AGL64*
Carubv10021725m.g	*AT1G29962*	*AGL64*
Carubv10021838m.g	*AT1G29962*	*AGL64*
Carubv10022052m.g	*AT1G29962*	*AGL64*
Carubv10022123m.g	*AT1G29962*	*AGL64*
Carubv10022431m.g	*AT1G29962*	*AGL64*
Carubv10028105m.g	*AT5G38620*	*AGL73*
Carubv10019521m.g	*AT1G48150*	*AGL74*
Carubv10006350m.g	*AT5G41200*	*AGL75*
Carubv10006654m.g	*AT5G41200*	*AGL75*
Carubv10007456m.g	*AT5G41200*	*AGL75*
Carubv10007591m.g	*AT5G41200*	*AGL75*
Carubv10015354m.g	*AT5G41200*	*AGL75*
Carubv10028378m.g	*AT5G65330*	*AGL78*
Carubv10014232m.g	*AT5G48670*	*AGL80, FEM111*
Carubv10028059m.g	*AT5G48670*	*AGL80, FEM111*
Carubv10027979m.g	*AT5G58890*	*AGL82*
Carubv10021267m.g	*AT1G54760*	*AGL85*
Carubv10011611m.g	*AT1G31630*	*AGL86*
Carubv10021558m.g	*AT1G54760*	*AGL86*
Carubv10006189m.g	*AT1G22590*	*AGL87*
Carubv10011303m.g	*AT1G22590*	*AGL87*
Carubv10021360m.g	*AT1G22590*	*AGL87*
Carubv10021540m.g	*AT1G22590*	*AGL87*
Carubv10021762m.g	*AT1G22590*	*AGL87*
Carubv10022252m.g	*AT1G22590*	*AGL87*
Carubv10001114m.g	*AT5G27960*	*AGL90*
Carubv10014761m.g	*AT3G66656*	*AGL91*
Carubv10003716m.g	*AT5G26950*	*AGL93*
Carubv10015395m.g	*AT2G15660*	*AGL95*
Carubv10002761m.g	*AT5G06500*	*AGL96*
Carubv10002823m.g	*AT5G06500*	*AGL96*
Carubv10022197m.g	*AT1G46408*	*AGL97*
Carubv10021917m.g	*AT5G04640*	*AGL99*
Carubv10025699m.g	*AT5G04640*	*AGL99*
Carubv10011075m.g	*AT1G17310*	*AGL100*
Carubv10011345m.g	*AT1G47760*	*AGL102*

Carubv10011458m.g	*AT1G47760*	*AGL102*
Carubv10012009m.g	*AT1G47760*	*AGL102*
Carubv10012603m.g	*AT1G47760*	*AGL102*
Carubv10024968m.g	*AT1G47760*	*AGL102*
Carubv10015986m.g	*AT3G18650*	*AGL103*
