# Supplementary material for: Non-reciprocal Interspecies Hybridization Barriers in the Capsella Genus Are Established in the Endosperm
Source: PLoS Genet. 2015 Jun 18;11(6):e1005295. doi: 10.1371/journal.pgen.1005295 (PMC4472357; doi:10.1371/journal.pgen.1005295)
Supplement: S5 Table — (PDF) [file pgen.1005295.s010.pdf]

S5 Table. Genetic models predicting segregation of hybrid incompatibility in crosses of *C. rubella* × F2 plants (derived after selfing of an F1 plant *C. grandiflora* × *C. rubella*). The first model is based on a negative genetic interaction of one *C. rubella* maternal locus with two *C. grandiflora* paternal loci, while the second model is based on a negative genetic interaction of one *C. rubella* maternal locus with three *C. grandiflora* paternal loci. Paternal loci are written A, B and C if inherited from *C. grandiflora* and a, b, c if inherited from *C. rubella*. *F2 survival rate depends on the presence of the *C. rubella* maternal locus, which for simplicity has not been indicated.

2 paternal *C. grandiflora* loci interacting with 1 *C. rubella* maternal locus

Genotype	Frequency of genotypes in F2	F2 survival rate*	Proportion of surviving F2	Relative frequency of surviving F2	Expected seed abortion rate in cross *C. rubella* × F2	Frequency of F2s giving rise to seed abortion
AABB	6.30%	50% survive	3.2%	3.6%	100%	3.6%
AAbB	12.50%	75% survive	9.4%	10.7%	50%	21.4%
aABB	12.50%	75% survive	9.4%	10.7%	50%	
aAbB	25%	87.5% survive	21.9%	25.0%	25%	25.0%
AAbb	6.30%	100% survive	6.3%	7.2%	0%	
aAbb	12.50%	100% survive	12.5%	14.3%	0%	
aaBB	6.30%	100% survive	6.3%	7.2%	0%	50.1%
aabB	12.50%	100% survive	12.5%	14.3%	0%	
aabb	6.30%	100% survive	6.3%	7.2%	0%	
		**Total**	**87.7%**	**100.0%**		

3 paternal *C. grandiflora* loci interacting with 1 *C. rubella* maternal locus

Genotype	Frequency of genotypes in F2	F2 survival rate*	Proportion of surviving F2	Relative frequency of surviving F2	Expected seed abortion rate in cross *C. rubella* × F2	Frequency of F2s giving rise to seed abortion
AABBCC	1.60%	50% survive	0.8%	0.9%	100%	0.9%
AABBcC	3.10%	75% survive	2.3%	2.5%	50%	
AAbBCC	3.10%	75% survive	2.3%	2.5%	50%	7.4%
aABBCC	3.10%	75% survive	2.3%	2.5%	50%	

AAbBcC	6.30%	87.5% survive	5.5%	5.9%	25%	
aABBcC	6.30%	87.5% survive	5.5%	5.9%	25%	17.6%
aAbBCC	6.30%	87.5% survive	5.5%	5.9%	25%	
aAbBcC	12.50%	93.75% survive	11.7%	12.5%	12.5%	12.5%
AABBcc	1.60%	100% survive	1.6%	1.7%	0%	
AAbBcc	3.10%	100% survive	3.1%	3.3%	0%	
aABBcc	3.10%	100% survive	3.1%	3.3%	0%	
aAbBcc	6.30%	100% survive	6.3%	6.7%	0%	
AAbbCC	1.60%	100% survive	1.6%	1.7%	0%	
AAbbcC	3.10%	100% survive	3.1%	3.3%	0%	
aAbbCC	3.10%	100% survive	3.1%	3.3%	0%	
aAbbcC	6.30%	100% survive	6.3%	6.7%	0%	
AAbbcc	1.60%	100% survive	1.6%	1.7%	0%	
aAbbcc	3.10%	100% survive	3.1%	3.3%	0%	61.7%
aaBBCC	1.60%	100% survive	1.6%	1.7%	0%	
aaBBcC	3.10%	100% survive	3.1%	3.3%	0%	
aabBCC	3.10%	100% survive	3.1%	3.3%	0%	
aabBcC	6.30%	100% survive	6.3%	6.7%	0%	
aaBBcc	1.60%	100% survive	1.6%	1.7%	0%	
aabBcc	3.10%	100% survive	3.1%	3.3%	0%	
aabbCC	1.60%	100% survive	1.6%	1.7%	0%	
aabbcC	3.10%	100% survive	3.1%	3.3%	0%	
aabbcc	1.60%	100% survive	1.6%	1.7%	0%	
		**Total**	**94.0%**	**100.0%**		
