# Supplementary material for: Non-reciprocal Interspecies Hybridization Barriers in the Capsella Genus Are Established in the Endosperm
Source: PLoS Genet. 2015 Jun 18;11(6):e1005295. doi: 10.1371/journal.pgen.1005295 (PMC4472357; doi:10.1371/journal.pgen.1005295)
Supplement: S6 Table — (PDF) [file pgen.1005295.s011.pdf]

S6 Table. Primer sequences used for qPCR.

Primer	Forward	Reverse
PP2A	5'- AAACTCTTGCCTGCGGTTAT -3'	5'- AGGTTCACTGCGAGCTTCCTCA -3'
PHE1	5'-CATCAAGAGAAGGGGCTGAG-3'	5'- GCGATCCTCTCACACAACA-3'
AGL36	5'- ATGTGGGATATGCTGGAGGA -3'	5'- GAAAGGCATGTGATTGGTGAT -3'
AGL28	5'- TGCTTCAGAACCAGTCAACG -3'	5'- CATTGAATCCAAACGGGAGA -3'
AGL61	5'- TGGGAAAAGCCAGTAGAGG -3'	5'- GGAGCCATATTTGCCACAAC -3'
AGL62	5'- ATGGACGATGGGAGTCGTAT-3'	5'- CAAACGGCAATGCATGACTA-3'
ADM	5'- TTGGAGACATCATTGGCAAC-3'	5'- TCATCGCTTTGACTGTCACC-3'
