# Supplementary material for: Non-reciprocal Interspecies Hybridization Barriers in the Capsella Genus Are Established in the Endosperm
Source: PLoS Genet. 2015 Jun 18;11(6):e1005295. doi: 10.1371/journal.pgen.1005295 (PMC4472357; doi:10.1371/journal.pgen.1005295)
Supplement: S7 Table — (PDF) [file pgen.1005295.s012.pdf]

S7 Table. Quality of sequencing samples. Replicates are biological replicates. *Cr*, *Capsella rubella*, *Cg* *Capsella grandiflora*.

Library	Mapped Reads	Average Coverage	Average Reads per Gene	Mapping Efficiency
Cr × *Cr* Rep1	69,225,933	45.04	1282	96%
Cr × *Cr* Rep2	51,777,128	34.81	937	89%
Cr × *Cg* Rep1	69,802,639	44.64	1292	95%
Cr × *Cg* Rep2	53,293,936	33.69	936	88%
Cg × *Cg* Rep1	61,373,924	38.65	1131	96%
Cg × *Cg* Rep2	56,617,904	36.05	1026	89%
Cg × *Cr* Rep1	61,471,676	41.45	1111	87%
Cg × *Cr* Rep2	54,225,770	35.24	970	91%
